# Procaspase activating compound 1 controls tetracycline repressor-regulated gene expression system

**DOI:** 10.1042/BSR20180793

**Published:** 2019-01-08

**Authors:** Chiman Song, Namkyoung Kim, Miri Park, Jiyeon Lee, Ki-Bong Oh, Taebo Sim

**Affiliations:** 1 Chemical Kinomics Research Center, Korea Institute of Science and Technology, 5 Hwarangro 14-gil, Seongbuk-gu, Seoul 02792, Republic of Korea; 2 Department of Agricultural Biotechnology, College of Agriculture and Life Sciences, Seoul National University, 1 Gwanak-ro, Gwanak-gu, Seoul 08826, Republic of Korea; 3 Insert Affiliation Text Here; 4 KU-KIST Graduate School of Converging Science and Technology, Korea University, 145 Anam-ro, Seongbuk-gu, Seoul 02841, Republic of Korea

**Keywords:** anti-induction, antagonist, gene regulation, PAC-1, tetracycline repressor, TetR-regulated system

## Abstract

The tetracycline repressor (TetR)-regulated system is a widely used tool to study gene functions through control of its expression. Various effectors such as tetracycline (Tc) and doxycycline (Dox) quickly induce or shut down gene expression, but reversing gene expression has not been eligible due to long half-lives of such effectors. Here, we found that procaspase activating compound 1 (PAC-1) rapidly reduces transient expression of TetR-regulated green fluorescent protein (GFP) in mammalian cells. Next, we applied PAC-1 to control of expression of transient receptor potential melastatin 7 (TRPM7) protein, whose downstream cellular events can be monitored by cell morphological changes. We observed that PAC-1 quickly reduces TRPM7 expression, consequently affecting cell morphology regulated by TRPM7. The present study demonstrates the first small molecule that efficiently turns off the TetR-regulated gene expression in mammalian cells, thereby precisely regulating the expression level of target gene.

## Introduction

Techniques for gene expression regulation are valuable tools to characterize genes in organisms. The tetracycline repressor (TetR)-regulated system is one of the most widely used techniques to control gene expression in both prokaryotes and eukaryotes [1–3], where gene expression is induced by effectors like tetracycline (Tc) and doxycycline (Dox) [4]. In detail, Tc induces expression of target gene by forcing dissociation of TetR from tetracycline operator (*tetO*) upon binding to TetR [5,6]. Numerous efforts including TetR modification [7,8], *tetO* specificity manipulation [9–11], and effector developments [12–16] have been made to improve the TetR-regulated system.

Fast on/off switches will enhance the capability of the TetR-regulated system to study biological roles of gene of interest in developmental processes. It is, however, yet challenging to achieve fast on/off switching due to the long half-life of Tc [17]. Tc-free medium or food has been used to switch off target gene expression by diluting Tc once it activates gene expression in cells or in animals. Although decrease in target gene expression was achieved by dilution, the dilution process is quite slow, taking even days [18–20]. To accomplish rapid turn-on/off of target gene expression, developing effectors acting like a Tc antagonist is required. TAP1, TCP1, and K79 peptides were discovered as Tc antagonists using the yeast two-hybrid method [13,16]. Goeke et al. have reported that TAP1 and TCP1 peptides were Tc antagonists interacting with TetR residues binding the C- or D-rings of Tc [13]. K79 peptide exerts its antagonistic activity in mammalian cells by binding into the Tc-binding site of the reverse transregulator, rtTA. A small molecule, GR33076X, is an antagonist acting on the transactivator, tTA in bacterial cells [12]. These are only four antagonists reported to date, so additional efforts are needed to discover novel molecules that rapidly switch on/off target gene expression.

Mg^2+^ plays a critical role in TetR/Tc complex formation [21]; Tc/Mg^2+^ complex dissociates TetR from TetR/*tetO* complex via its strong interaction with TetR [22]. We, thus, hypothesized that removal of Mg^2+^ by a metal chelator would restore interactions between TetR and *tetO*, thereby suppressing transcription of target gene. Given that membrane-permeability is essential for a metal chelator to act in cells, we tested procaspase activating compound 1 (PAC-1), a known membrane-permeable metal chelator, and discovered PAC-1 quickly turns off TetR-regulated green fluorescent protein (GFP) expression. Furthermore, we showed that PAC-1 exerts its antagonistic effect on TetR-regulated transient receptor potential melastatin 7 (TRPM7) protein expression in the stable expression system. We also demonstrated that cell morphological changes controlled by TRPM7 can be regulated by PAC-1.

## Materials and methods

### Antibodies and reagents

Doxycycline hyclate, MG-132, and TPEN (*N*,*N*,*N*′,*N*′-tetrakis(2-pyridylmethyl)ethylenediamine) were purchased from Sigma-Aldrich (U.S.A.) and PAC-1 was purchased from Tocris Bioscience (U.K.). Antibodies against GFP (B-2), β actin (8H10D10), Fas (CH11), TRPM7 (N74/25), and poly(ADP-ribose) polymerase-1 (PARP-1, SC-7150) were purchased from Santa Cruz Biotechnology (U.S.A.), Cell Signalling Technology (U.S.A.), Millipore (Germany), NeuroMab (U.S.A.), and Santa Cruz Biotechnology (U.S.A.), respectively. DNA oligomers were purchased from Cosmogenetech (ROK). K79 peptide (RVDDAMTPRMWHGLIF) was synthesized by Anygen (ROK).

### Cell culture and preparation

T-REx-293 cells stably expressing Dox-inducible TRPM7 via the inducible TetR(B)-regulated system [23] were kindly provided by Prof. Byung Joo Kim (Pusan National University, ROK). T-REx-293 cells expressing TRPM7 were cultured in Dulbecco’s modified Eagle’s media (DMEM) supplemented with 10% fetal bovine serum (FBS), 100 U/ml penicillin, 100 μg/ml streptomycin, 5 μg/ml blasticidin, and 0.5 mg/ml zeocin in a humidified 5% CO_2_ incubator at 37°C. T-REx-293 cells (Life Technologies, U.S.A.) were maintained in DMEM with 10% FBS, 100 U/ml penicillin, 100 μg/ml streptomycin, and 15 μg/ml blasticidin. All cells were passaged every 2 or 3 days.

### Molecular biology

A TetR(B)-regulated GFP construct was generated by inserting GFP cDNA as a *BamH*I–*BamH*I fragment into the pcDNA5/FRT/TO vector (Life Technologies, U.S.A.). GFP cDNA was amplified using forward and reverse primers; 5′-TACCGAGCTCGGATCCATGGTGAGCAAGGGCGAG-3′ and 5′-CTGGACTAGTGGATCCTTACTTGTACAGCTCGTC-3′. TetR(B) cDNA from pcDNA6/TR (Invitrogen, U.S.A.) was amplified using forward and reverse primers; 5′-AAGGAGATATACATATGTCTAGATTAGATAAAAG-3′ and 5′-GGTGGTGGTGCTCGAGGGACCCACTTTCACATTTAAG-3′, and inserted as a *Nde*I-*Xho*I fragment into the pET-21b(+) vector (Novagen/Merck, Germany) to generate a pET-21b(+)-TetR(B) construct. All constructs were verified by DNA sequencing (Cosmogenetech, ROK).

### Reverse transcription PCR analysis

T-REx-293 cells were seeded on 100 mm dishes (Thermo, U.S.A.) at 2.0 × 10^6^ cells per dish, and transfected with pcDNA5/FRT/TO-GFP using Lipofectamine 2000 (Life Technologies, U.S.A.). After transfection, T-REx-293 cells transiently expressing GFP were seeded on 60 mm dishes at 8.0 × 10^5^ cells per dish, and incubated with or without 1 μM Dox in the absence or presence of PAC-1 (10 and 100 μM) for 2 and 8 h at 37°C in a humidified 5% CO_2_ incubator. For T-Rex-293 cells stably expressing Dox-inducible TRPM7, the cells were seeded on 60 mm dishes at 8.0 × 10^5^ cells per dish, and incubated in the absence or presence of PAC-1 (10 and 100 μM) in the absence of Dox for 4 h after induction with 1 μM Dox for 4 h. Total RNA was extracted using TRIZOL (Invitrogen, U.S.A.). Reverse transcription PCR (RT-PCR) reactions were performed with 1 μg of total RNA using Maxime™ RT PreMix (Intron Biotechnology, ROK). For quantitative comparisons, PCR reactions were carried out using HiPi Plus PCR Premix (Elpis, ROK) with forward and reverse primers to β-actin; 5′-TCCTGTGGCATCCACGAAACT-3′ and 5′-GAAGCATTTGCGGTGGACGAT-3′. For GFP RT-PCR, GFP cloning primers were used. For TRPM7 RT-PCR, forward and reverse primers, 5′-CCATACCATATTCTCCAAGGTTCC-3′ and 5′-CATTCCTCTTCAAATCTGGAAGTT-3′, respectively, were used. PCR products were resolved on 1.8% agarose gels and relative mRNA levels were determined by densitometry analysis using Image J (National Institutes of Health, U.S.A.).

### Western blot analysis

T-REx-293 cells were transfected with pcDNA5/FRT/TO-GFP as described above. T-REx-293 cells transiently expressing GFP were seeded on 60 mm dishes at 8.0 × 10^5^ cells per dish, and incubated with or without 1 μM Dox in the absence or presence of PAC-1 (10 and 100 μM) for 2, 4, and 8 h at 37°C in a humidified 5% CO_2_ incubator. Cells were, then, washed twice with Dulbecco’s phosphate-buffered saline (PBS; WelGene, ROK), and lysed in RIPA buffer (CellNest, China) with the Protease Inhibitor Cocktail Solution (Sigma-Aldrich, U.S.A.) for 30 min at 4°C. Proteins from cell lysates were quantified using the BCA Assay Kit (Thermo/Pierce, U.S.A.), and equivalent amounts of proteins were loaded on 7.5% sodium dodecylsulfate polyacrylamide gels (SDS-PAGE). Separated proteins were transferred to a 0.20 μm polyvinylidene fluoride (PVDF) membrane (Millipore, Germany), and the membrane was blocked with 5% skim milk in Tris-buffered saline Tween-20 (137 mM NaCl, 20 mM Tris, 0.1% Tween-20, and pH 7.4) for 2 h. After blocking, the membrane was incubated with primary antibodies overnight at 4°C, and horseradish peroxidase (HRP)-conjugated anti-mouse IgG (Cell Signalling Technology, U.S.A.) was used as a secondary antibody. Complex with HRP-linked secondary antibody was detected using the ECL Substrate Kit (Thermo/Pierce, U.S.A.). Densitometry analysis of Western blot data was carried out using Image J.

T-REx-293 cells expressing TRPM7 were seeded on 60 mm dishes at 8.0 × 10^5^ cells per dish, and incubated in the absence or presence of PAC-1 (1, 3, 10, 30, and 100 μM) in the absence of Dox for 1, 2, and 4 h after induction with 1 μM Dox for 4 h. T-REx-293 cells expressing TRPM7 were incubated with or without 1 μM Dox for 8 h in the presence of 1 μg/ml anti-Fas for apoptosis stimulation or 10 μM MG-132 for proteasome inhibition. For TPEN, T-REx-293 cells expressing TRPM7 were incubated in the absence or presence of TPEN (1, 3, 10, 30, and 100 μM) in the absence of Dox for 4 h after induction with 1 μM Dox for 4 h. Western blot analysis was performed described above. The concentration–response curve was fitted to a Hill equation to obtain the half-maximal inhibitory concentration (IC_50_).

### Cell rounding analysis

T-REx-293 cells expressing TRPM7 were seeded on 60 mm dishes at 8.0 × 10^5^ cells per dish, and incubated with PAC-1 (0, 10, and 100 μM) in the absence of Dox for 4 h after treatment with 1 μM Dox for 4 h. Cell images were taken using an Eclipse *Ti* microscope (Nikon, Japan). Degree of cell roundness was evaluated according to previous method [24]. Fully rounded cells without membrane extension, partially rounded cells with one or two membrane extensions, and non-rounded cells with three or four membrane extensions were given one point, half a point, and zero point, respectively.

### Flow cytometric analysis

Flow cytometric analysis was performed using the Apoptosis Detection Kit with FITC-labeled annexin V and propidium iodide (PI) (BioLegend, U.S.A.). T-REx-293 cells expressing TRPM7 were seeded on 60 mm dishes at 8.0 × 10^5^ cells per dish, and incubated for 24 h at 37°C in a humidified 5% CO_2_ incubator. After removing culture media, 1 μg/ml Dox in 5 ml fresh media was added to each dish to induce TRPM7 expression. After induction for 4 h, cells were incubated with PAC-1 (0, 10, and 100 μM) in the absence of Dox for 4 h. After incubation, cells were harvested, washed with PBS, and resuspended in Annexin V Binding Buffer. Cells were stained with FITC-labeled annexin V for 15 min at room temperature in the dark, followed by addition of PI. Stained cells were immediately analyzed using BD FACS Canto II (BD Biosciences, U.S.A.).

### Ultraviolet–visible absorption spectra analysis

Absorption spectra of PAC-1 were obtained at 0, 10, 19, 27, 34, 42, 48, 55, 61, 66, 71, and 76 μM in assay solution (50 mM Hepes, 100 mM KNO_3_, and pH 7.2) using DU-650, a ultraviolet–visible (UV–vis) spectrophotometer (Beckman Coulter, U.S.A.). The molar extinction coefficients of PAC-1 (ε_PAC-1_) were determined at 282 and 323 nm from the slope of linear regression fitting. The molar extinction coefficients of PAC-1/Mg^2+^ complex (${\text{ε}}_{\text{PAC-}1/{\text{Mg}}^{2+}}$) were also determined at 282 and 323 nm by obtaining absorption spectra with PAC-1 at 0, 10, 19, 27, 34, 42, 48, 55, 61, 66, 71, and 76 μM in the presence of 1.5 M Mg^2+^ at its saturating concentration in assay solution. Concentrations of free PAC-1 ([PAC-1]_free_) were calculated using ε_PAC-1_. Concentrations of PAC-1/Mg^2+^ complex ([PAC-1/Mg^2+^]) and free Mg^2+^ ([Mg^2+^]_free_) were calculated using the following equations [25]:

$$\text{Absorbance}={\text{ε}}_{\text{PAC-}1}{\left[\text{PAC-}1\right]}_{\text{total}}+\left({\text{ε}}_{\text{PAC-}1/{\text{Mg}}^{2+}}-{\text{ε}}_{\text{PAC-}1}\right)\left[\text{PAC-}1/{\text{Mg}}^{2+}\right]$$

$${\left[{\text{Mg}}^{2+}\right]}_{\text{free}}={\left[{\text{Mg}}^{2+}\right]}_{\text{total}}-\left[\text{PAC-}1/{\text{Mg}}^{2+}\right]$$

where [PAC-1]_total_ and [Mg^2+^]_total_ are total concentration of PAC-1 and Mg^2+^, respectively.

The equilibrium dissociation constant (*K*_D_) of PAC-1/Mg^2+^ complex was determined by measuring absorbance at 282 and 323 nm at 0, 0.5, 1, 2, 5, 10, 21, 50, 101, 202, and 499 mM Mg^2+^ in the presence of 75 μM PAC-1 in assay solution. *K*_D_ was obtained by fitting a fractional saturation curve to a saturation binding model with one site including total and nonspecific binding.

### Expression and purification of TetR protein

*Escherichia coli* BL21 (DE3) cells were transformed with pET-21b(+)-TetR plasmid. Cells were grown in LB broth supplemented with 50 μg/ml ampicillin at 37°C on a 250 rpm shaking incubator, and protein expression was induced with 0.2 mM isopropyl β-d-thiogalactopyranoside (IPTG) at OD_600_ of 0.7. Induced cells were incubated for 6 h at 26°C, harvested by centrifugation at 3000 rpm for 10 min, washed with phosphate buffer (50 mM sodium phosphate, 100 mM NaCl, 5 mM MgCl_2_, 1 mM EDTA, 1 mM DTT, and pH 7.0), and re-suspended in phosphate buffer. Cell suspension was lysed by ultra-sonication on ice. Cell debris and unbroken cells were removed by centrifugation at 15000 rpm for 30 min, and the supernatant was loaded onto the nickel-nitrilotriacetic acid (Ni-NTA) affinity column. Proteins were eluted with a linear gradient of imidazole (0.1−1 M) in phosphate buffer. Fractions were collected and analyzed by SDS-PAGE. TetR containing fractions were pooled, dialyzed with 50% glycerol in phosphate buffer to remove imidazole, and stored in aliquots at −80°C until use. The protein concentration was determined using the BCA Assay Kit (Thermo/Pierce, U.S.A.).

### Surface plasmon resonance analysis

The interaction between TetR and PAC-1 was quantified using surface plasmon resonance (SPR) analysis with the Reichert SR7500DC SPR system (Reichert Technologies, U.S.A.). TetR was immobilized on a carboxymethyl dextran hydrogel (CMDH) surface sensor chip using 1-ethyl-3-(3-dimethylaminopropyl) carbodiimide hydrochloride (EDC) and *N*-hydroxysuccinimide (NHS). PAC-1 was injected over the sensor chip at 10, 20, 40, 80, 160, 320, and 640 μM in HEPES buffer (100 mM NaCl, 2 mM MgCl_2_, 10 mM HEPES, and pH 7.5) at a rate of 30 μl/min. Binding affinity between TetR and PAC-1 was measured as response unit (RU). RU was recorded for 6 min in every injection, and the background response recorded from a reference flow cell was subtracted from each sample. Data were analyzed using Scrubber2 software (Biologic Software, Australia). *K*_D_ was determined by the ratio of dissociation to association rate constants (*k*_d_/*k*_a_).

### Electrophoretic mobility-shift assay

*tetO* was formed by annealing 5′-CCTAATTTTTGTTGACACTCTATCATTGATAGAGTTATTTTACCACTC-3′ and 5′-GAGTGGTAAAATAACTCTATCAATGATAGAGTGTCAACAAAAATTAGG-3′. For DNA hybridization, equal molar amounts of each oligomer were mixed, heated for 5 min at 96°C, and cooled down to RT. DNA was incubated with various concentrations of TetR, Dox, and PAC-1 in binding buffer (20 mM Tris-HCl, 50 mM NaCl, 1 mM MgCl_2_, 0.1 mM DTT, and pH 8.0) for 15 min at room temperature. After incubation, DNA was resolved on 8% polyacrylamide gels in electrophoresis buffer (90 mM Tris, 90 mM boric acid, and 5 mM MgCl_2_). For detection, DNA was stained with ethidium bromide.

### Data analysis

All data were analyzed using Prism 6 software (GraphPad, U.S.A.), and presented as mean ± s.e.m. Statistical significance was evaluated by one-way ANOVA with Tukey’s multiple comparison test. Statistical values of *P*<0.05 were considered to be statistically significant.

## Results

### PAC-1 turns off TetR-regulated GFP expression

**Figure 1:**
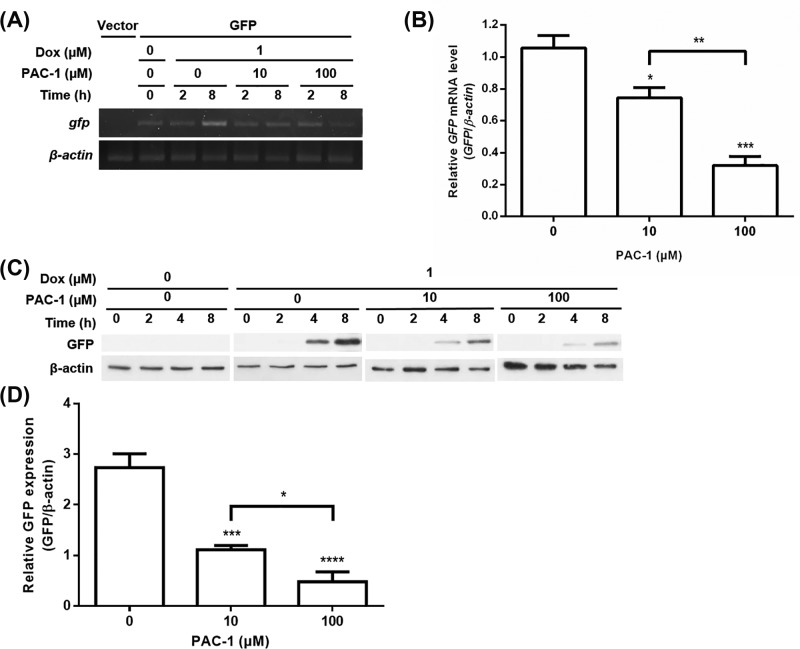
PAC-1 turns off TetR-regulated GFP expression (**A**) Time course of GFP mRNA expression obtained from RT-PCR analysis. T-REx-293 cells transfected with pcDNA5/FRT/TO-GFP were incubated with PAC-1 at 0, 10, and 100 µM in the presence of 1 µM Dox for 0, 2, and 8 h. (**B**) Densitometry analysis of RT-PCR data with PAC-1 (0, 10, and 100 µM) at 8 h. (**C**) Time course of GFP protein expression obtained from Western blot analysis. T-REx-293 cells transiently expressing GFP were incubated with PAC-1 at 0, 10, and 100 µM in the presence of 1 µM Dox for 0, 2, 4, and 8 h. (**D**) Densitometry analysis of Western blot data with PAC-1 (0, 10, and 100 µM) at 8 h. All densitometry data were normalized to the intensity of β-actin bands. Error bars represent s.e.m. (*n*=3). Statistical significance was evaluated by one-way ANOVA with Tukey’s multiple comparison tests; ^*^P<0.05, ^**^P<0.01, ^***^P<0.001, and ^****^P<0.0001.

To assess effects of PAC-1 on the TetR-regulated system, a *gfp* reporter gene controlled by TetR was used. GFP mRNA expression levels were measured by RT-PCR analysis at 0, 2, and 8 h after simultaneous treatment with 1 μM Dox and PAC-1 at 0, 10, and 100 μM (Figure 1A). GFP mRNA levels were reduced by PAC-1 in a concentration-dependent manner at 8 h (Figure 1B), showing 29% and 70% decrease by 10 and 100 μM PAC-1, respectively. Residual GFP mRNA expression was observed in the absence of Dox, which may be due to Tc contamination in FBS. To further evaluate PAC-1 effects at the protein level, GFP protein expression was monitored by Western blot analysis at 0, 2, 4, and 8 h after simultaneous treatment with 1 μM Dox and PAC-1 at 0, 10, and 100 μM (Figure 1C). GFP expression was detected at 4 h after induction, and time-dependent increase in GFP expression was observed in all Dox-treated cells. TetR-regulated GFP protein expression levels were decreased by 59% and 82% in the presence of 10 and 100 μM PAC-1, respectively (Figure 1D). These results demonstrate that PAC-1 is capable of switching off TetR-regulated GFP expression in the transient expression system.

### PAC-1 controls cellular events through regulation of TetR-regulated TRPM7 expression

**Figure 2:**
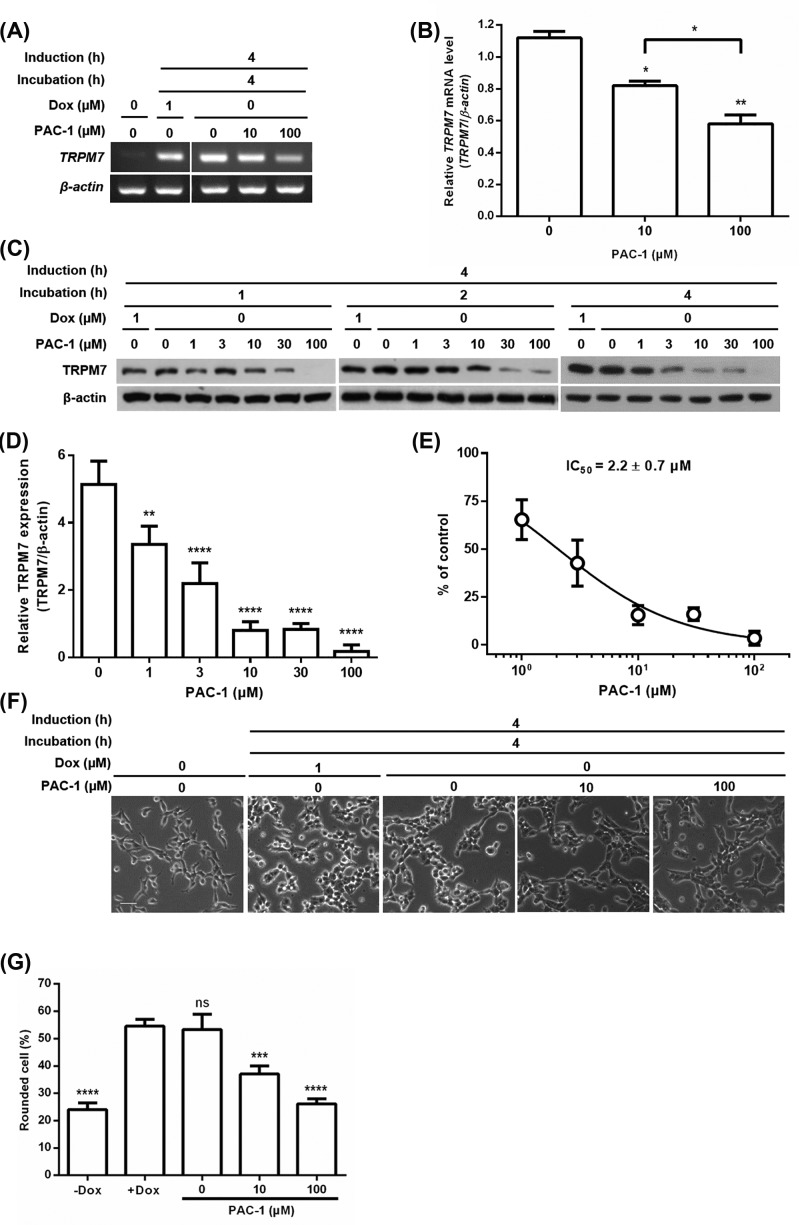
PAC-1 rescues cell rounding by switching off TetR-regulated TRPM7 expression (**A**) Time course of TRPM7 mRNA expression obtained from RT-PCR analysis. T-REx-293 cells stably expressing Dox-inducible TRPM7 were incubated with PAC-1 at 0, 10, and 100 µM in the absence of Dox for 4 h after induction with 1 µM Dox for 4 h. (**B**) Densitometry analysis of RT-PCR data in (A). (**C**) Time course of TRPM7 protein expression obtained from Western blot analysis. T-REx-293 cells stably expressing Dox-inducible TRPM7 were incubated with PAC-1 at 0, 1, 3, 10, 30, and 100 µM in the absence of Dox for 1, 2, and 4 h after induction with 1 µM Dox for 4 h. (**D**) Densitometry data normalized to the intensity of β-actin bands at 4 h. (**E**) The dose–response curve of PAC-1 generated from densitometry data in (D). (**F**) Representative microscopic images of T-REx-293 cells stably expressing Dox-inducible TRPM7. Cells were incubated with PAC-1 at 0, 10, and 100 µM in the absence of Dox for 4 h after induction with 1 µM Dox for 4 h. Scale bar represents 50 µm. (**G**) Quantification of the degree of cell roundness in (F). Error bars represent s.e.m. (*n*=3). Statistical significance was evaluated by one-way ANOVA with Tukey’s multiple comparison tests; ^*^P<0.05, ^**^P<0.01, ^***^P<0.001, ^****^P<0.0001, and ns = not significant.

Ectopic expression of TRPM7 via TetR-regulated gene expression system caused cell rounding [26], so we further investigated whether PAC-1 can control TRPM7-mediated cell rounding by turning off TetR-regulated gene expression using T-REx-293 cells stably expressing Dox-inducible TRPM7 [24,26]. To examine effects of PAC-1 on the TetR-regulated TRPM7 mRNA expression level, RT-PCR analysis was performed in the presence of PAC-1 (10 and 100 μM) in the absence of Dox for 4 h after induction with 1 μM Dox for 4 h (Figure 2A). PAC-1 reduced TRPM7 mRNA levels in a concentration-dependent manner (Figure 2B), showing 27% and 48% decrease by 10 and 100 μM PAC-1, respectively. To evaluate effects of PAC-1 on the TetR-regulated TRPM7 protein level, its expression was monitored by Western blot analysis with various concentrations of PAC-1 (0, 1, 3, 10, 30, and 100 μM) at 1, 2, and 4 h after induction with 1 μM Dox for 4 h (Figure 2C). Densitometry analysis showed that TRPM7 expression is suppressed by PAC-1 in a dose-dependent manner at 4 h (Figure 2D). Its IC_50_ value of 2.2 ± 0.7 μM was determined from the concentration–response curve generated using densitometry data at 4 h (Figure 2E). Similarly, K79 peptide as a Tc antagonist suppressed Dox-inducible TRPM7 expression in a dose-dependent fashion, but it reduced the TRPM7 expression by only 59.64 ± 1.34% even at 100 μM (Supplementary Figures S1A and S1B). Based on the densitometry data, PAC-1 was more effective than K79 in antagonistic effect. Furthermore, we tested whether PAC-1 can control cell rounding induced by TRPM7 [24,26]. Morphological changes of T-REx-293 cells stably expressing Dox-inducible TRPM7 were monitored by an inverted microscope with or without PAC-1 (10 and 100 μM) at 4 h after induction with 1 μM Dox for 4 h (Figure 2F,G). While cells were rounded over 50% after treatment of 1 μM Dox for 4 h, roundness of cells treated with PAC-1 remarkably reduced, suggesting that the level of protein expression regulated by PAC-1 is correlated with the degree of protein-induced cell phenotype. Overall, these results imply that PAC-1 can be utilized to modulate cellular events via protein expression regulation.

To further confirm the possibility that antagonistic effect of PAC-1 could be happen under maximum Dox-induced protein expression, we monitored TetR-regulated TRPM7 protein expression in T-REx-293 cells stably expressing Dox-inducible TRPM7 in the presence of PAC-1 under maximum TRPM7 protein expression. First of all, to find proper induction time for maximum TRPM7 protein expression, we observed its expression level over time after induction with Dox (Supplementary Figure S2A). After induction with Dox for 24 h, TRPM7 protein was maximally expressed (Supplementary Figure S2B). Next, we investigated how long maximum TRPM7 protein expression took to reach baseline following removal of Dox. After induction with Dox for 24 h, T-REx-293 cells stably expressing Dox-inducible TRPM7 was incubated in the absence of Dox. It was not until 96 h that TRPM7 protein expression level was decreased to baseline (Supplementary Figure S2C). However, PAC-1 significantly reduced Dox-inducible TRPM7 protein level to baseline in just 4 h (Supplementary Figure S2D). Moreover, re-induction with Dox restored Dox-inducible TRPM7 protein level reduced by PAC-1 (Supplementary Figure S2E). These results support that antagonistic effect of PAC-1 could happen under maximum protein expression induced by Dox and is reversible.

### Proteasomal activity of PAC-1 is partially involved in its antagonistic effect

**Figure 3:**
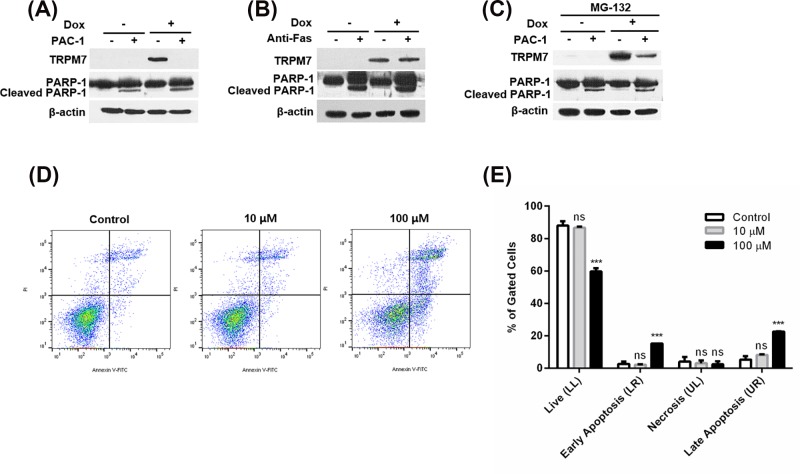
Proteasomal activity of PAC-1 is partially involved in its antagonistic effect TRPM7 protein expression in the presence of 100 µM PAC-1 (**A**) or 1 µg/ml anti-Fas, an apoptosis inducer (**B**). T-REx-293 cells stably expressing Dox-inducible TRPM7 were incubated in the absence or presence of 1 µM Dox for 8 h with 100 µM PAC-1 or with 1 µg/ml anti-Fas. (**C**) T-REx-293 cells expressing TRPM7 were incubated with 100 µM PAC-1 in the absence or presence of 1 µM Dox in the presence of 10 µM MG-132 (proteasome inhibitor) for 8 h. (**D**) T-REx-293 cells stably expressing Dox-inducible TRPM7 were incubated with PAC-1 at 0, 10, and 100 µM for 4 h after induction with 1 µM Dox for 4 h. Apoptosis was analyzed by annexin V-FITC/PI staining. (**E**) Percentages of gated cells in each quadrant. LL, LR, UL, and UR are lower left, lower right, upper left, and upper right quadrants, respectively. Error bars represent s.e.m. (*n*=3). Statistical significance was evaluated by one-way ANOVA with Tukey’s multiple comparison tests; ^***^P<0.001 and ns = not significant.

PAC-1 was initially discovered as an apoptosis inducer activating procaspase-3 to caspase-3 [27]. We, thus, examined whether TetR-regulated TRPM7 expression levels are affected by caspase-dependent apoptosis. To confirm PAC-1-induced apoptosis, a cleaved PARP-1 fragment as a prominent apoptotic marker was detected by Western blot analysis (Figure 3A). TetR-regulated TRPM7 expression was completely diminished by 100 μM PAC-1 in the presence of 1 μM Dox at 8 h (Figure 3A), suggesting that PAC-1 reduces TetR-regulated TRPM7 expression. In contrast with PAC-1, anti-Fas antibody as an apoptosis inducer did not reduce TRPM7 expression (Figure 3B), indicating that apoptotic activity of PAC-1 is not responsible for its antagonistic effect on TetR-regulated TRPM7 expression. Next, we tested whether proteasome-dependent protein degradation pathway contributes to decrease in TRPM7 expression by treatment of PAC-1 using MG-132 (proteasome inhibitor) because PAC-1 promotes the proteasome-dependent degradation pathway [28]. TRPM7 expression was merely detected after co-treatment with 1 μM Dox and 100 μM PAC-1 in the presence of 10 μM MG-132 (Figure 3C). Inhibition of proteasome activity could slightly reverse suppression of TetR-regulated TRPM7 expression by PAC-1, indicating that its proteasomal activity is involved in its antagonistic effects, but not a major cause of the effects.

We also tested whether apoptotic activity of PAC-1 limits its usage as a Tc antagonist. To evaluate apoptotic activity of PAC-1, we performed flow cytometric analysis with annexin V-FITC and PI (Figure 3D,E). Percentage of gated cells in apoptosis stages was increased significantly in the presence of 100 μM PAC-1 for 4 h, while PAC-1 did not induce the apoptosis of T-REx-293 cells expressing TRPM7 at 10 μM. This observation is consistent with the previous finding that PAC-1 induces apoptosis [27]. These results suggest that PAC-1 could be used at low concentrations as an antagonist without affecting cellular apoptosis although it has apoptosis-inducing activity.

### PAC-1 has a lower affinity for Mg^2+^ and TetR than Dox

**Figure 4:**
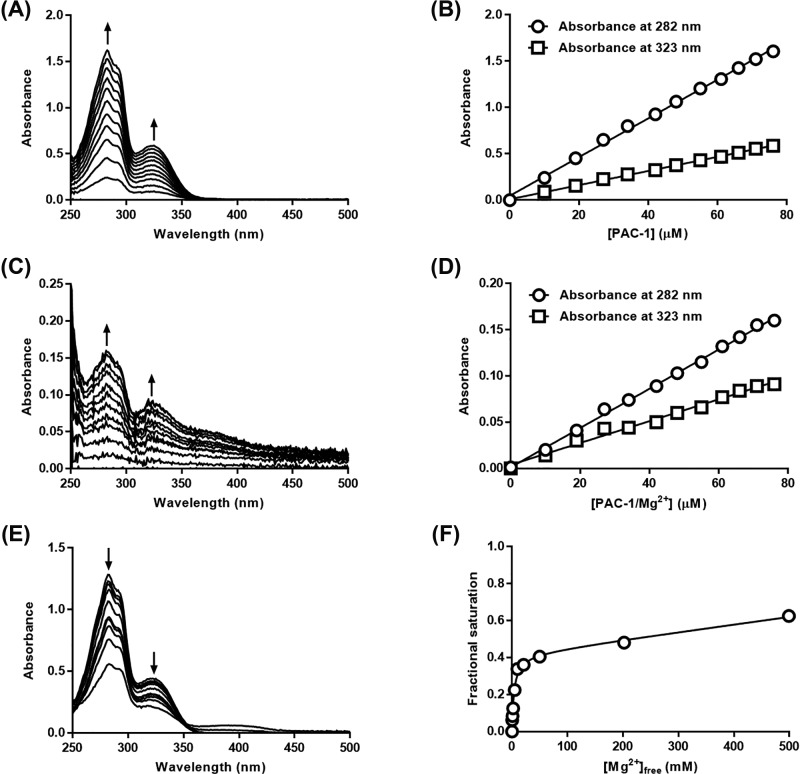
Binding affinity of PAC-1 to Mg^2+^ is lower than that of Dox (**A**) Absorption spectra of PAC-1 at various concentrations (0−76 μM). (**B**) The molar extinction coefficients of PAC-1 at 282 and 323 nm were determined as a slope from linear regression fitting. (**C**) Absorption spectra of PAC-1/Mg^2+^ complex at various concentrations of PAC-1 (0−76 μM) in the presence of 1.5 M Mg^2+^ at its saturating concentration. (**D**) The molar extinction coefficients of PAC-1/Mg^2+^ complex at 282 and 323 nm were determined as a slope from linear regression fitting. (**E**) PAC-1 titration with 0−499 mM Mg^2+^. (**F**) Fractional saturation curve of PAC-1/Mg^2+^ complex generated from (E) was fitted to a saturation binding model with one site including total and nonspecific binding. The arrows in (A), (C), and (E) indicate the peak wavelengths at 282 nm and 323 nm.

Mg^2+^ is required for strong interactions between TetR and Dox [21], and PAC-1 is a known divalent cation chelator [29]. We, thus, hypothesized that PAC-1 exerts its antagonistic effect by chelating Mg^2+^. To test this hypothesis, we determined *K*_D_ of PAC-1/Mg^2+^ complex using the UV–vis absorption spectroscopic method. Absorption spectra of PAC-1 exhibited the two distinctive peaks at 282 and 323 nm (Figure 4A), and its molar extinction coefficients were determined as 0.0225 and 0.0076 μM^−1^cm^−1^ at 282 and 323 nm, respectively (Figure 4B). The molar extinction coefficients of PAC-1/Mg^2+^ complex were also determined from its absorption spectra measured at various concentrations of PAC-1 (0−76 μM) in the presence of 1.5 M Mg^2+^ at its saturating concentration (Figure 4C), resulting in 0.0021 and 0.0012 μM^−1^ cm^−1^ at 282 and 323 nm, respectively (Figure 4D). *K*_D_ of PAC-1/Mg^2+^ complex was determined by titrating 75 μM PAC-1 with 0−499 mM Mg^2+^ (Figure 4E). The fractional saturation curve generated using Mg^2+^ titration data provided *K*_D_ of 4.0 ± 0.8 mM (Figure 4F), which is 10-fold higher than *K*_D_ of Dox/Mg^2+^ complex, 0.4 mM [15].

**Figure 5:**
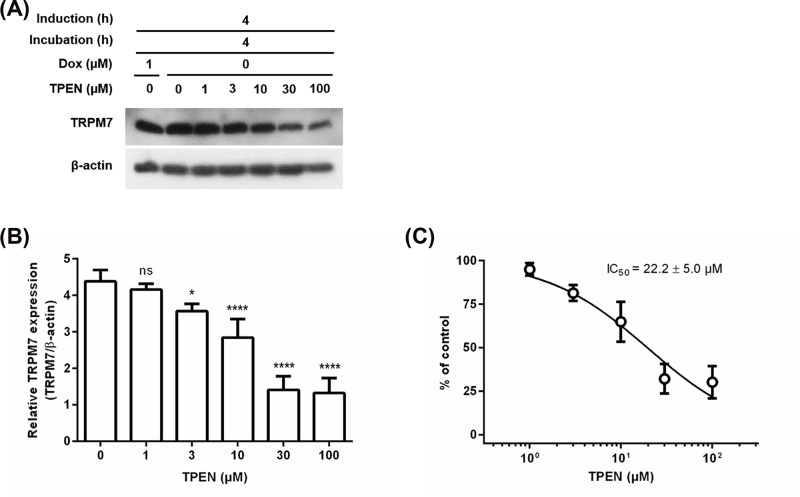
TPEN decreases TetR-regulated TRPM7 expression (**A**) TRPM7 protein expression obtained from Western blot analysis. T-REx-293 cells stably expressing Dox-inducible TRPM7 were incubated with TPEN at 0, 1, 3, 10, 30, and 100 µM in the absence of Dox for 4 h after induction with 1 µM Dox for 4 h. (**B**) Densitometry data normalized to the intensity of β-actin bands. Error bars represent s.e.m. (*n=3*). Statistical significance was evaluated by one-way ANOVA with Tukey’s multiple comparison tests; ^*^P <0.05, ^****^P <0.0001, and ns = not significant. (**C**) The dose–response curve of TPEN generated from densitometry data in (B).

To further confirm the hypothesis that antagonistic effect of PAC-1 might be associated with Mg^2+^ chelation, we assessed TetR-regulated TRPM7 protein expression in T-REx-293 cells stably expressing Dox-inducible TRPM7 in the presence of different concentrations (0, 1, 3, 10, 30, and 100 μM) of TPEN, a metal chelator (Figure 5A). TPEN suppressed TRPM7 expression in a dose-dependent manner (Figure 5B), and Its IC_50_ value (22.2 ± 5.0 μM) determined from the concentration–response curve (Figure 5C) is approximately 10-fold higher than that of PAC-1. Affinity of TPEN for Mg^2+^ (*K*_D_ = 20 mM) [30], which is 5-fold higher than that of PAC-1 for Mg^2+^, may contribute to the difference in the antagonistic effect between PAC-1 and TPEN. These results showed that TPEN could also play as an antagonist in the TetR-regulated system and metal chelation might be required for the antagonistic effect.

**Figure 6:**
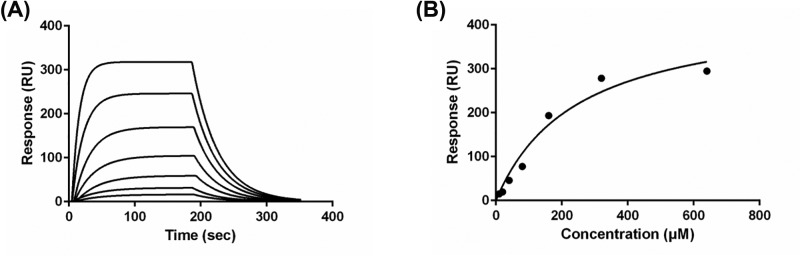
SPR analysis of interaction between PAC-1 and TetR (**A**) Sensogram from the titration of TetR with PAC-1. The interaction between TetR and PAC-1 was quantified using SPR analysis. PAC-1 was injected onto the TetR-immobilized sensor chip at 10, 20, 40, 80, 160, 320, and 640 μM. Binding affinity between TetR and PAC-1 was measured as RU. (**B**) Saturation curve of TetR responses as a function of PAC-1 concentration. The obtained data were further analyzed to extract association and dissociation rate constants (*k*_a_ and *k*_d_). *K*_D_ of TetR/PAC-1 complex was calculated from its kinetic parameters (*k*_d_/*k*_a_).

Next, we tested the possibility of direct binding of PAC-1 to TetR as an underlying mechanism for switching off TetR-regulated gene expression. To measure a binding affinity of PAC-1 to TetR, we performed SPR analysis using recombinant TetR protein. TetR was immobilized on a sensor chip, and PAC-1 was added onto the chip at 10, 20, 40, 80, 160, 320, and 640 μM (Figure 6A). The obtained sensogram was further analyzed to extract association and dissociation rate constants: *k*_a_ = 98 ± 2 M^−1^ s^−1^ and *k*_d_ = 0.0258 ± 0.0002 s^−1^ (Figure 6B). *K*_D_ of TetR/PAC-1 complex calculated from its kinetic parameters (*k*_d_/*k*_a_) is 2.6 x 10^−4^ M, which is 7 orders of magnitude higher than that of TetR/Dox complex (*K*_D_ of 4.4 x 10^−11^ M) [15]. Taken together, we conclude that direct interaction with TetR would not be a main mechanism accounting for the antagonistic effect of PAC-1 on the TetR-regulated system.

**Figure 7:**
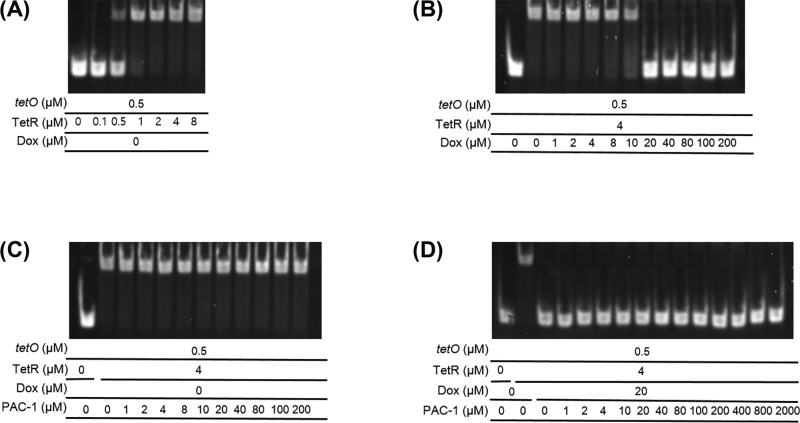
PAC-1 does not alter associations of TetR with both Dox and *tetO* (**A**) TetR-dependent TetR/*tetO* complex formation in the presence of 0.5 μM *tetO*. (**B**) Dox-dependent TetR/*tetO* complex dissociation in the presence of 0.5 μM *tetO* and 4 μM TetR. (**C**) No alteration of TetR/*tetO* complex by PAC-1 up to 0.2 mM in the absence of Dox. (**D**) No alteration of TetR/Dox complex by PAC-1 up to 2 mM in the presence of 20 μM Dox based on the intact *tetO* oligomer bands. Upper bands indicate TetR/*tetO* complex and lower bands indicate free *tetO* oligomers.

Finally, we tested effects of PAC-1 on TetR/*tetO* and TetR/Dox complexes using electrophoretic mobility-shift assay (EMSA) with recombinant TetR protein and *tetO* oligomers. The upper bands indicating TetR/*tetO* complex in Figure 7 start appearing at 0.5 μM TetR in the presence of 0.5 μM *tetO* (Figure 7A), and they completely disappear by 20 μM Dox (Figure 7B) in the presence of 4 μM TetR and 0.5 μM *tetO* as expected. However, PAC-1 showed no effect either on TetR/*tetO* complex (upper bands) up to 0.2 mM in the absence of Dox (Figure 7C), or on *tetO* oligomers (lower bands), presumably TetR/Dox complex up to 2 mM in the presence of 20 μM Dox (Figure 7D). These results indicate that PAC-1 does not affect both TetR/*tetO* and TetR/Dox complexes.

## Discussion

The TetR-regulated system is a valuable tool to control gene expression both *in vitro* and *in vivo* [1–3]. Although numerous effectors have been developed for this system [12–16], only four effectors have been reported to reverse Tc-induced gene expression [12,13,16]. GR33076X, a Tc homolog is a small-molecule antagonist acting on tTA in bacterial cells, but its effect was shown only at 100- to 1000-fold higher concentration than Tc [12]. Although TAP1 and TCP1 as TetR-regulating peptides have antagonistic activities in the presence of non-saturating amount (0.03 μM) of Dox [13], usage of the peptides is inconvenient for the TetR-regulated system because transformation of plasmids harboring the peptides and addition of IPTG are needed to express them in cells. Schmidt et al. reported K79, which is a peptide switching off gene expression mediated by rtTA, but its effect was apparent only after treatment with it for 10 h [16]. Thus, antagonists with a better efficacy and a faster mode-of-action need to be developed for the TetR-regulated system.

Here, we discovered PAC-1 is a novel small-molecule antagonist for the TetR-regulated system. We demonstrated that PAC-1 regulates TetR-regulated gene expression in both transient and stable expression systems. When the concentration of PAC-1 was 10-fold higher than that of Dox, it showed significant antagonistic effects. In addition, PAC-1 quickly exerts its antagonistic effect even at 4 h after treatment and usage of PAC-1 is so much easier than that of the previously reported peptides because it is a small molecule. Overall, these properties are better than those of previously reported antagonists such as GR33076X and K79. Furthermore, we observed that protein expression levels controlled by PAC-1 have a positive correlation with the degree of cell morphological changes induced by TetR-regulated TRPM7 expression. We, thus, believe that PAC-1 would be a useful tool to study physiological roles of protein through accurate tuning of protein expression levels.

Although PAC-1 has been previously shown apoptosis-inducing activity in several cancer cell lines [27,28] at concentrations less than or equal to 10 μM, it did not induce the apoptosis of T-REx-293 cells expressing TRPM7 at 10 μM concentration in the present study. This might result from different susceptibility of cell lines to PAC-1. Putt et al. reported that PAC-1 induces cell death in several cancer cell lines in a procaspase-3 dependent fashion, and primary cancerous colon cells having elevated concentrations of procaspase-3 are more susceptible to PAC-1 than noncancerous cells having relatively low concentrations of that [27]. Thus, T-REx-293 cells as a noncancerous cell might be less susceptible to PAC-1 than cancer cell lines. PAC-1 exhibited its antagonistic effect even at 10 μM in TetR-regulated TRPM7 expression (Figure 2C), and it sufficiently rescued cell phenotype controlled by TetR-regulated TRPM7 at 10 μM (Figure 2F,G). Taken together, we suggest that PAC-1 could be utilized as an antagonist for the TetR-regulated system without affecting cellular apoptosis at low concentrations. Eventually, however, the issue needs to be resolved by exploring new PAC-1 analogs with no apoptotic activity.

To investigate the molecular mechanisms of PAC-1 as a Tc antagonist, we tested whether apoptotic activity of PAC-1 causes its antagonistic effect. We eliminate this possibility, because anti-Fas antibody-induced apoptosis did not reproduce the antagonistic effect of PAC-1. In contrast with apoptotic activity of PAC-1, its proteasomal activity is partially involved in the antagonistic effect. Next, we examined whether PAC-1 is an effective Mg^2+^ chelator because Mg^2+^ is important for formation of TetR/Dox complex [21]. However, *K*_D_ of PAC-1/Mg^2+^ complex was 10-fold higher than that of Dox/Mg^2+^ complex. Although Mg^2+^ chelation of PAC-1 would not be sufficient to demonstrate the antagonistic effect, we cannot overlook the effect of metal chelation on it because TPEN as a metal chelator also showed the antagonistic activity. We also tested a possibility for the direct interaction of PAC-1 with TetR using SPR analysis. *K*_D_ of TetR/PAC-1 complex was approximately 10^7^-fold higher than that of TetR/Dox complex. We, thus, rule out the direct binding mechanism. Additionally, we did not observe any effects of PAC-1 on both TetR/Dox and TetR/*tetO* complexes in EMSA experiments. Taken together, it appears that PAC-1 does not directly affect Dox-induced dissociation of TetR/*tetO* complex despite of its apparent antagonistic effects. It could be due to differences between *in vitro* and *in vivo* systems or presence of other metal-associated factors affecting TetR-regulated protein expression system. Further investigations would be required to identify its mode of actions. Nonetheless, the present study would provide the basis for the development of next-generation effectors that can accurately regulate TetR-regulated protein expression by quickly turning on/off gene expression.

## Supporting information

**Supplementary Figure S1.** K79 peptide suppresses Dox-inducible TRPM7 protein expression.

**Supplementary Figure S2.** Antagonistic effect of PAC-1 can happen under maximum TRPM7 protein expression.

## Abbreviations

DMEM: Dulbecco’s modified Eagle’s media
Dox: doxycycline
EMSA: electrophoretic mobility-shift assay
FBS: fetal bovine serum
GFP: green fluorescent protein
HRP: horseradish peroxidase
IC_50_: half-maximal inhibitory concentration
IPTG: isopropyl β-d-thiogalactopyranoside
*k*_d_/*k*_a_: ratio of dissociation to association rate constants
KD: equilibrium dissociation constant
[Mg^2+^]free: concentration of free Mg^2+^
εPAC-1: molar extinction coefficients of PAC-1
εPAC-1/Mg^2+^: molar extinction coefficients of PAC-1/Mg^2+^ complex
PAC-1: procaspase activating compound 1
[PAC-1]free: concentration of free PAC-1
[PAC-1/Mg^2+^]: concentration of PAC-1/Mg^2+^ complex
PBS: phosphate-buffered saline
PI: propidium iodide
RT-PCR: reverse transcription PCR
RU: response unit
SDS-PAGE: sodium dodecylsulfate polyacrylamide gels
SPR: surface plasmon resonance
Tc: tetracycline
tetO: tetracycline operator
TetR: tetracycline repressor
TPEN: *N,N,N*′,*N*′-tetrakis(2-pyridylmethyl)ethylenediamine
TRPM7: transient receptor potential melastatin 7
UV–vis: ultraviolet–visible
