# Supplementary material for: Procaspase activating compound 1 controls tetracycline repressor-regulated gene expression system
Source: Biosci Rep. 2019 Jan 8;39(1):BSR20180793. doi: 10.1042/BSR20180793 (PMC6328932; doi:10.1042/BSR20180793)
Supplement: Supplementary file 1 [file bsr20180793_Supp1.pdf]

Supplementary Material

Procaspase activating compound 1 controls tetracycline repressor-regulated gene expression system

Chiman Song[1,2], Namkyoung Kim[3], Miri Park[1], Jiyeon Lee[1], Ki-Bong Oh[2,*], Taebo Sim[1,3,*]

Supplementary Figure S1. K79 peptide suppresses Dox-inducible TRPM7 protein expression.

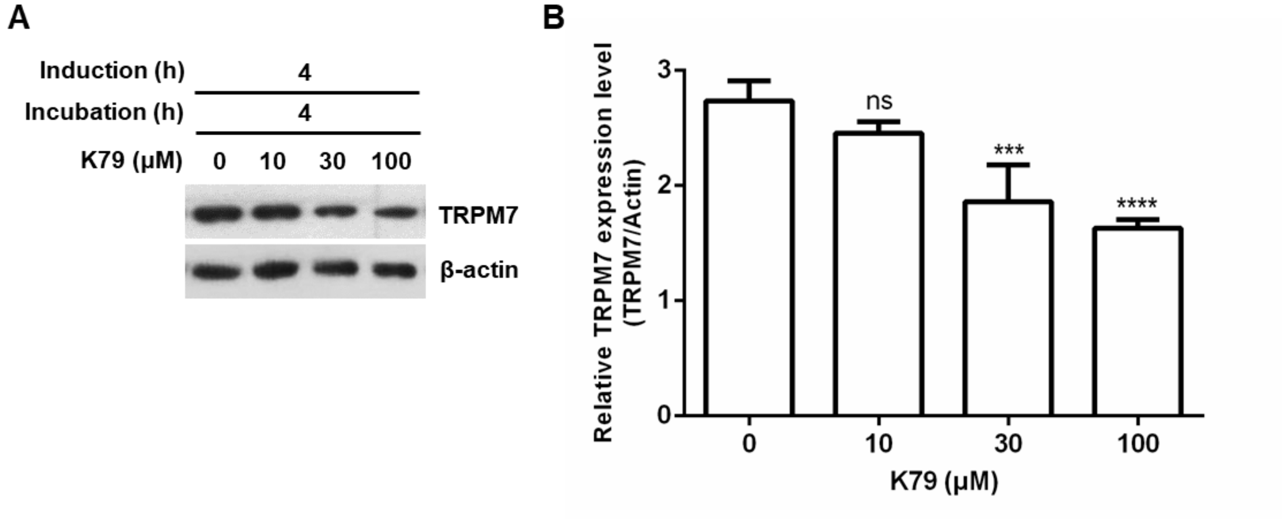

(A) Time course of TRPM7 protein expression obtained from Western blot analysis. T-REx-293 cells stably expressing Dox-inducible TRPM7 were incubated with K79 at 10, 30, and 100 µM for 4 h in the absence of Dox after induction with 1 µM Dox for 4 h. (B) Densitometric analysis of Western blot data in (A). Error bars represent s.e.m. (n = 3). Statistical significance was evaluated by one-way ANOVA with Tukey's multiple comparison tests; ***P <0.001, ****P <0.0001, and ns = not significant.

Supplementary Figure S2. Antagonistic effect of PAC-1 can happen under maximum TRPM7 protein expression.

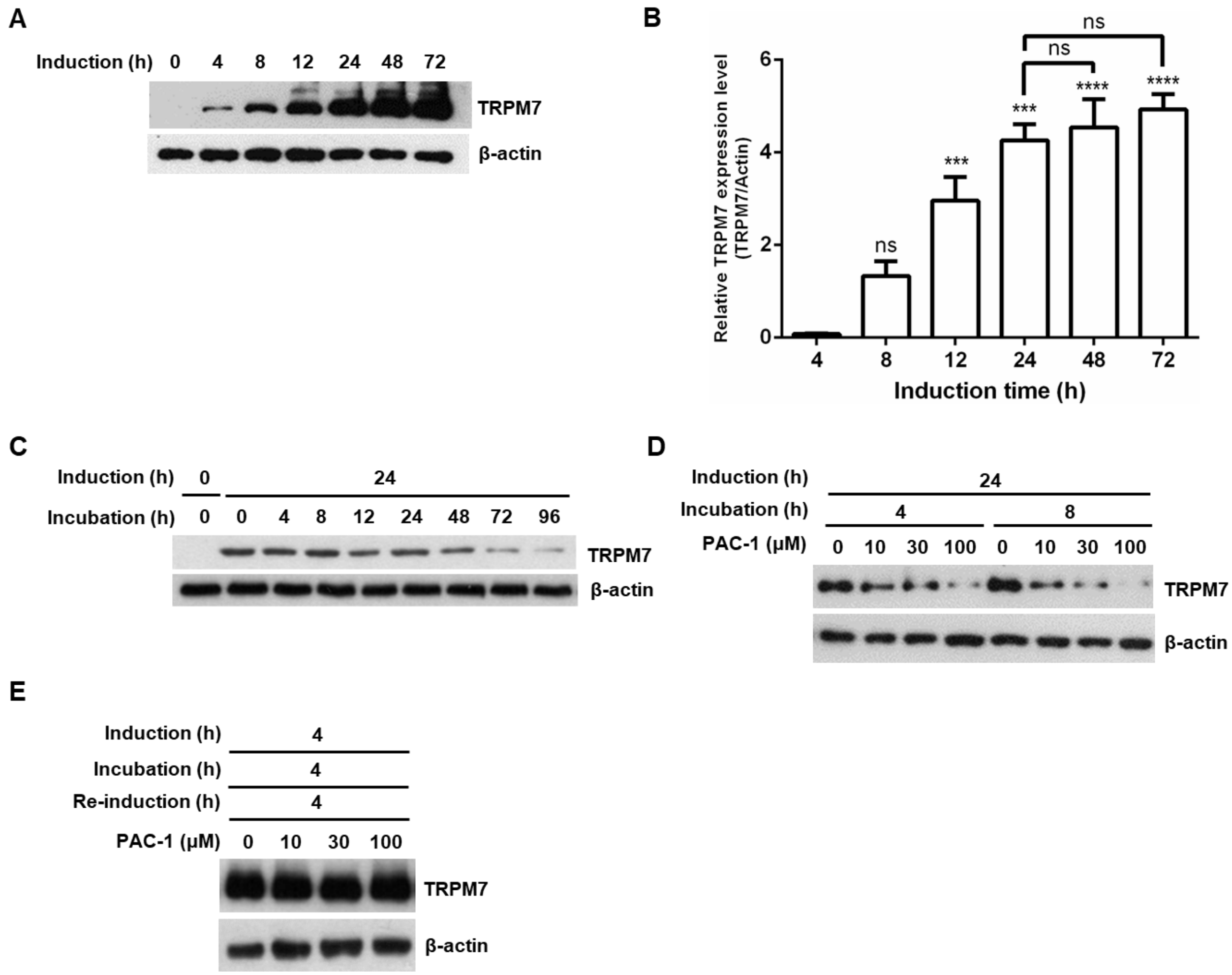

(A) Time course of TRPM7 protein expression obtained from Western blot analysis. T-REx-293 cells stably expressing Dox-inducible TRPM7 were incubated for 4, 8, 12, 24, 48, and 72 h in the presence of 1 μM Dox. (B) Densitometric analysis of Western blot data in (A). (C) T-REx-293 cells stably expressing Dox-inducible TRPM7 were incubated for 4, 8, 12, 24, 48, 72, and 96 h in the absence of Dox after induction with 1 μM Dox for 24 h. (D) T-REx-293 cells stably expressing Dox-inducible TRPM7 were incubated with PAC-1 at 10, 30, and 100 μM in the absence of Dox for 4 and 8 h after induction with 1 μM Dox for 24 h. (E) T-REx-293 cells stably expressing Dox-inducible TRPM7 were re-incubated with 1 μM Dox for 4 h following treatment of PAC-1 in the absence of Dox for 4 h after induction with 1 μM Dox for 4 h. Error bars represent s.e.m. (n = 3). Statistical significance was evaluated by one-way ANOVA with Tukey's multiple comparison tests; ***P <0.001, ****P <0.0001, and ns = not significant.
